# Supplementary material for: Genetic background and mistranslation frequency determine the impact of mistranslating tRNASerUGG
Source: G3 (Bethesda). 2022 May 19;12(7):jkac125. doi: 10.1093/g3journal/jkac125 (PMC9258585; doi:10.1093/g3journal/jkac125)
Supplement: jkac125_Supplemental_Material [file jkac125_supplemental_material.pdf]

Genetic background and mistranslation frequency determine the impact of mistranslating tRNA$^{Ser}_{UGG}$

Matthew D. Berg[1,†,‡], Yanrui Zhu[1], Raphaël Loll-Krippleber[2], Bryan-Joseph San Luis[2], Julie Genereaux[1], Charles Boone[2], Judit Villen[3], Grant W. Brown[2] and Christopher J. Brandl[1,†]

[1]Department of Biochemistry, The University of Western Ontario, London, Canada
[2]Donnelly Centre for Cellular and Biomolecular Research, University of Toronto, Toronto, Canada
[3]Department of Genome Sciences, University of Washington, Seattle, WA 98195, USA

[†]Co-corresponding author: Matthew Berg and Christopher Brandl, Department of Biochemistry, The University of Western Ontario, London, Ontario N6A 5C1, Canada, Email: mberg2@uwo.ca; cbrandl@uwo.ca
‡Present address: Department of Genome Sciences, University of Washington, Seattle, WA 98195, USA

Supplemental Material

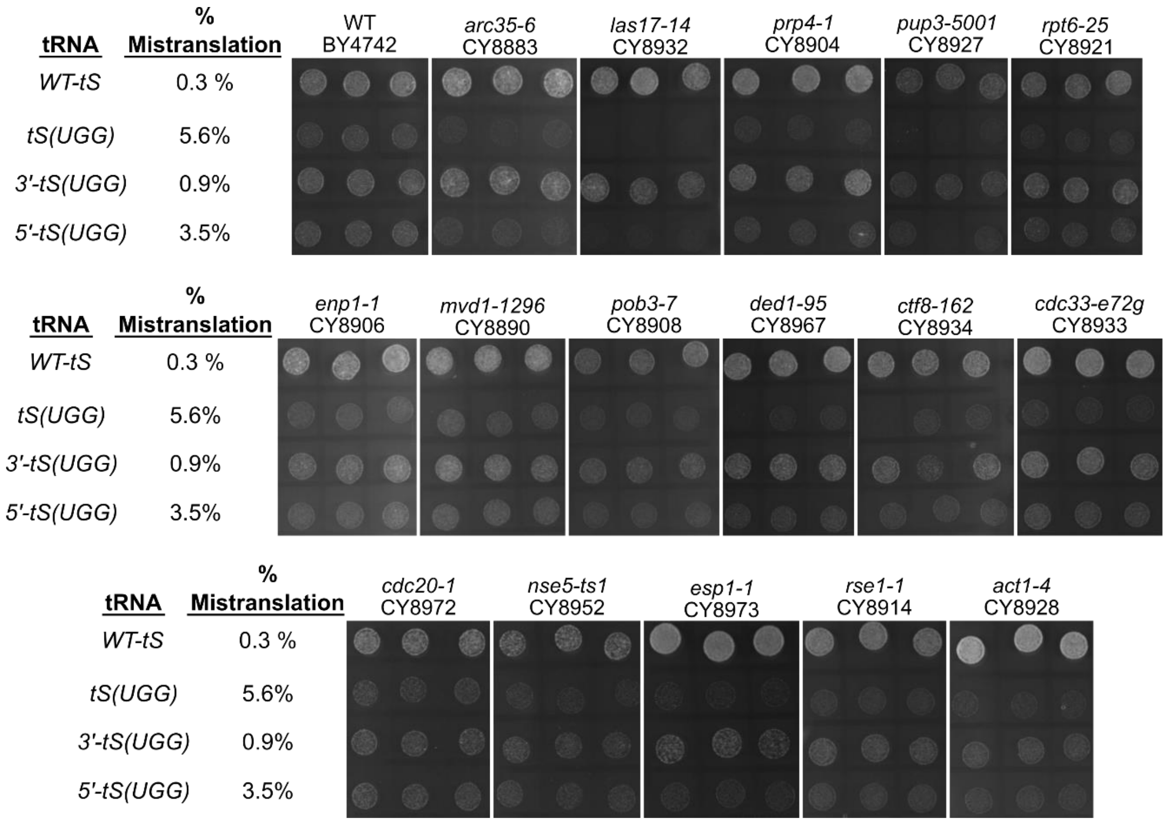

Figure S1. *Severity of different mistranslation frequencies differs depending on strain background.* Wild-type BY4742 or the indicated strains from the temperature sensitive collection were transformed with a *URA3* centromeric plasmid expressing either wild-type tRNA^{Ser} [*WT-tS*], tRNA$^{Ser}_{UGG,G26A}$-*GAL1pr* [*3'-tS(UGG)*], *GAL1pr*-tRNA$^{Ser}_{UGG,G26A}$ [*5'-tS(UGG)*] and tRNA$^{Ser}_{UGG,G26A}$ [*tS(UGG)]*. Strains were grown to confluency in media lacking uracil. Cells were diluted 33-fold and spotted on media lacking uracil with galactose as the carbon source. Each spot represents one biological replicate.

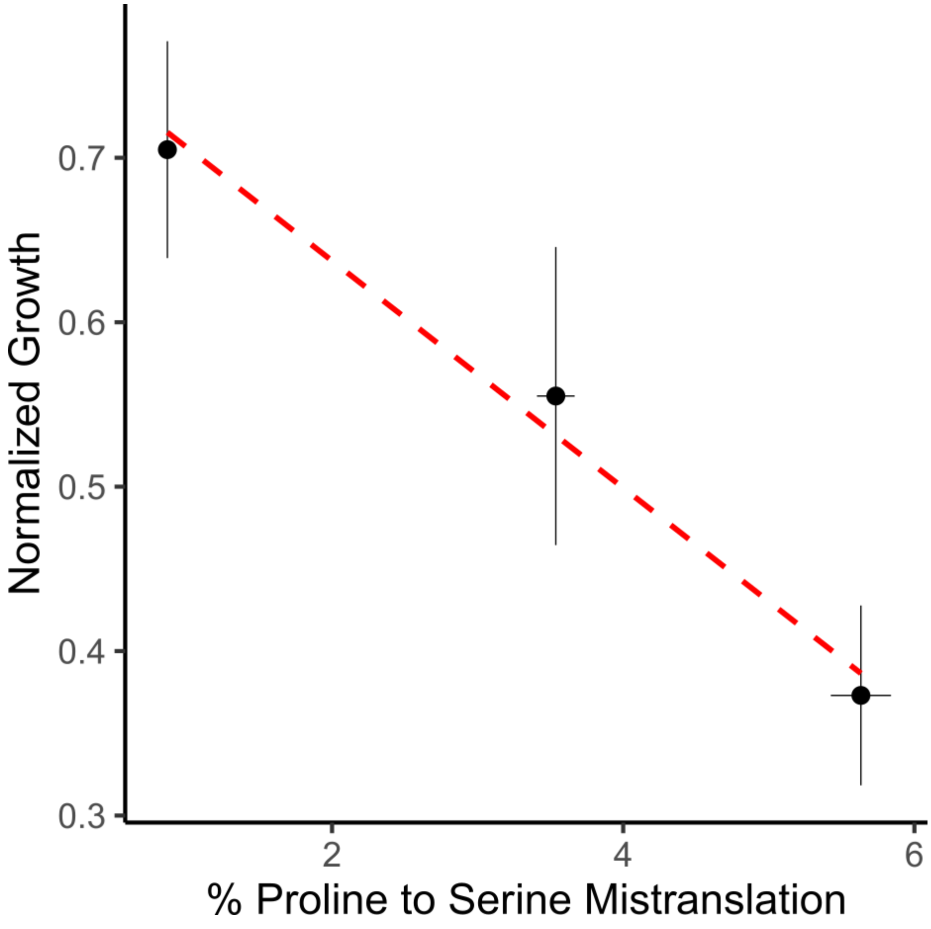

Figure S2. *Mistranslation frequency negatively correlates with growth.* Wild-type BY4742 was transformed with a *URA3* centromeric plasmid expressing either wild-type $tRNA^{Ser}$ [*WT-tS*], $tRNA^{Ser}_{UGG,G26A}$-*GAL1pr* [*3'-tS(UGG)*], *GAL1pr*-$tRNA^{Ser}_{UGG,G26A}$ [*5'-tS(UGG)*] and $tRNA^{Ser}_{UGG,G26A}$ [*tS(UGG)]*. Strains were grown to confluency in media lacking uracil. Cells were diluted 33-fold and spotted on media lacking uracil with galactose as the carbon source. The spot intensity of the strain containing the mistranslating tRNA was divided by the intensity of the strain containing the wild-type $tRNA^{Ser}$ to determine normalized growth. Whole proteome mass spectrometry was performed to determine percent proline to serine substitution in Berg *et al.* (2021b). Each point represents an average of 3 biological replicates and bars indicate the standard deviation. Red line is a linear regression fit to the data.

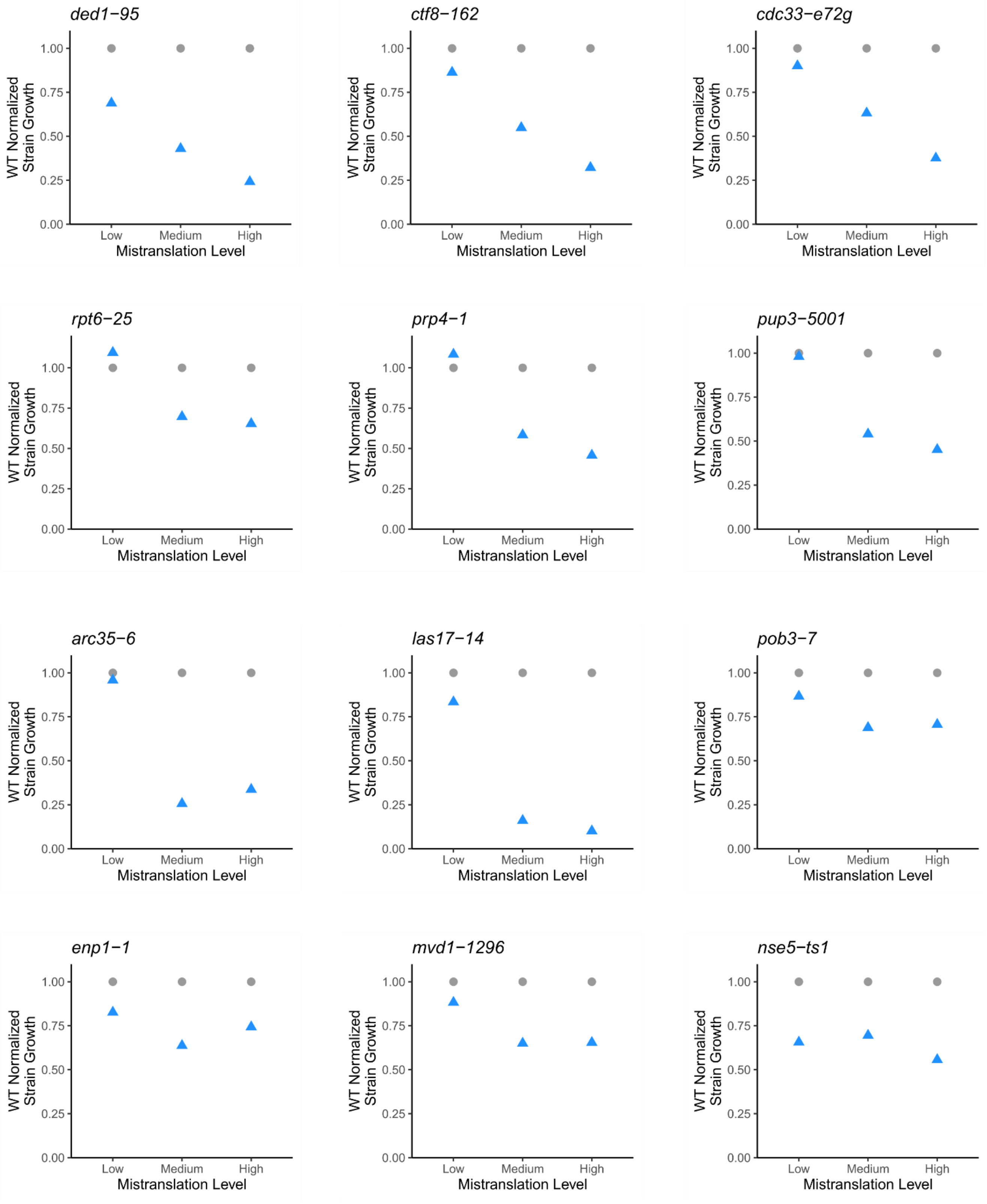
ded1−95
ctf8−162
cdc33−e72g
rpt6−25
prp4−1
pup3−5001
arc35−6
las17−14
pob3−7
enp1−1
mvd1−1296
nse5−ts1
WT Normalized Strain Growth
0.00 0.25 0.50 0.75 1.00
Low Medium High
Mistranslation Level

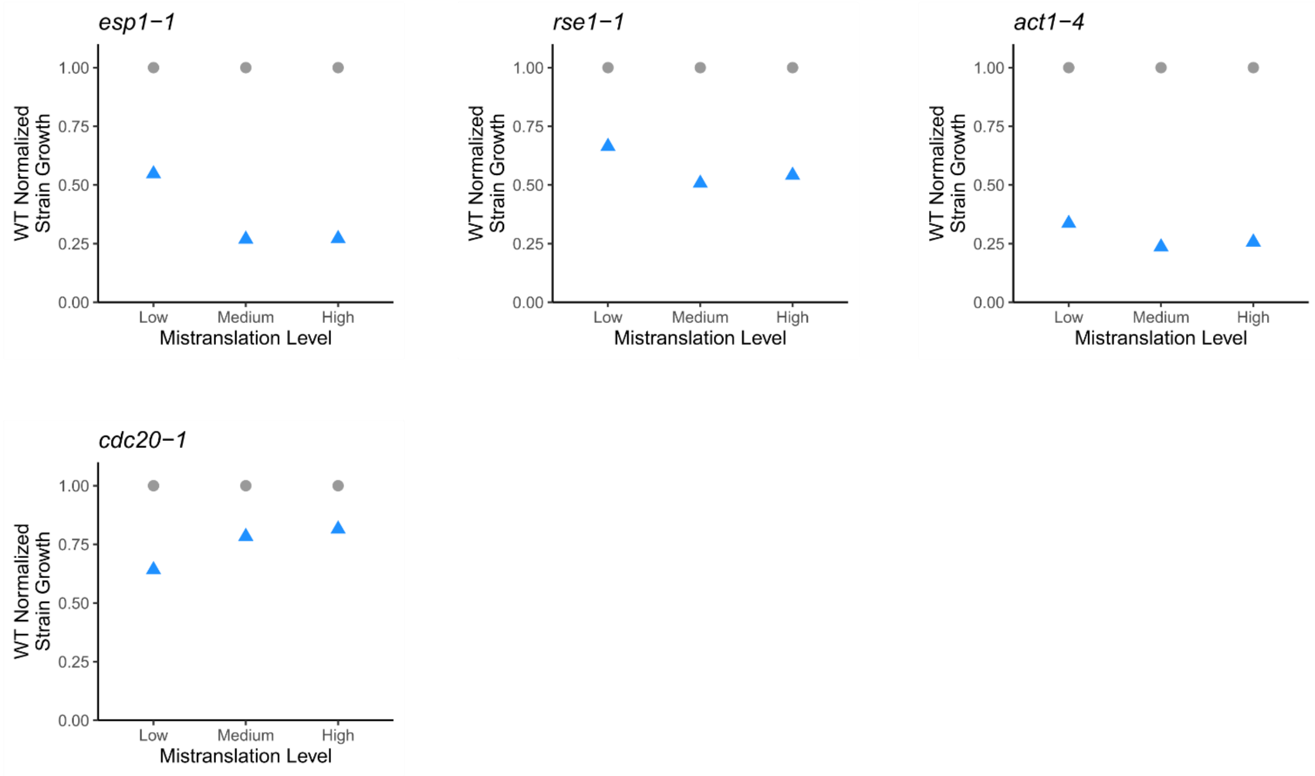

Figure S3. *Genetic background alters the impact of different frequencies of proline to serine mistranslation.* The average normalized growth calculated as in Figure 3 expressed as a percentage of the growth of the wild-type strain (BY4742) is shown in blue for the three different mistranslating constructs (Low: tRNA$^{Ser}_{UGG,G26A}$-*GAL1pr* [*3'-tS(UGG)*], Medium: *GAL1pr*-tRNA$^{Ser}_{UGG,G26A}$ [*5'-tS(UGG)*] and High: tRNA$^{Ser}_{UGG,G26A}$ [*tS(UGG)]*) for the temperature sensitive strains indicated. The growth of the wild-type strain, 100%, is plotted as grey dots. Each point is the average of at least three biological replicates as in Figure 3.
